# Reversible Systolic Anterior Motion in the Absence of Left Ventricular Hypertrophy Following Acute Myocardial Infarction: A Report of a Rare Case

**DOI:** 10.7759/cureus.102450

**Published:** 2026-01-28

**Authors:** Rima Chaddad, Maher Hakim, Virginie Carreira, Jacinthe Khater

**Affiliations:** 1 Department of Cardiology, Grand Hopital de l'Est Francilien, Paris, FRA; 2 Faculty of Medical Sciences, Lebanese University Faculty of Medicine, Beirut, LBN

**Keywords:** apical akinesia, dynamic obstruction, hcm mimic, left ventricular outflow tract obstruction, mitral regurgitation, myocardial infarction, pci, sam, systolic anterior motion

## Abstract

Systolic anterior motion (SAM) of the mitral valve is classically associated with hypertrophic cardiomyopathy (HCM); however, it may rarely occur in the absence of left ventricular hypertrophy (LVH), particularly in the setting of acute myocardial ischemia. This phenomenon remains poorly understood and poses diagnostic and therapeutic challenges.

We report the case of a 54-year-old woman presenting with non-ST-elevation myocardial infarction (NSTEMI). Transthoracic echocardiography showed a non-dilated left ventricle (LV end-diastolic diameter 45 mm), preserved systolic function (left ventricular ejection fraction (LVEF) 63%; end-diastolic volume 115 mL, end-systolic volume 42 mL), apical akinesia, and compensatory basal hyperkinesis. There was no LV hypertrophy (interventricular septum 8 mm; lateral wall 7 mm). SAM of the mitral valve was present, resulting in moderate mitral regurgitation and a dynamic left ventricular outflow tract (LVOT) gradient of 30 mmHg. The right ventricle was normal in size and function.

Cardiac magnetic resonance imaging demonstrated apical myocardial edema on T2-weighted sequences and a punctiform transmural late gadolinium enhancement in the inferomedial segment on phase-sensitive inversion recovery (PSIR) sequences, with no evidence of cardiomyopathy or structural substrate that could explain LVOT obstruction. Coronary angiography revealed a significant mid-left anterior descending artery lesion, successfully treated with drug-eluting stent implantation. Medical management included beta-blocker therapy and careful volume optimization, with avoidance of inotropes. Follow-up transesophageal echocardiography at three weeks confirmed complete resolution of SAM, mitral regurgitation, LVOT gradient, and apical wall-motion abnormalities. This case illustrates a rare, ischemia-induced and reversible form of SAM without LVH. LVH was excluded based on echocardiographic wall thickness measurements below established thresholds (interventricular septum 8 mm and lateral wall 7 mm), well under the diagnostic cutoff for LVH (>11 mm), likely mediated by transient apical dysfunction and basal hyperkinesis, altering ventricular geometry and flow dynamics.

## Introduction

Systolic anterior motion (SAM) of the mitral valve is most commonly associated with hypertrophic cardiomyopathy (HCM), where asymmetric septal hypertrophy and altered mitral valve anatomy contribute to dynamic left ventricular outflow tract (LVOT) obstruction [1]. However, the occurrence of SAM in the absence of left ventricular hypertrophy (LVH) is a rare and less-understood phenomenon. In such cases, SAM may be transient and reversible, often observed in the acute setting of myocardial infarction (MI), particularly when associated with apical wall-motion abnormalities and preserved or hyperdynamic basal function. Several pathophysiological mechanisms have been proposed to explain this atypical presentation. One leading hypothesis suggests that apical akinesia or hypokinesia during acute MI may result in compensatory basal hyperkinesis. This abnormal motion pattern can generate increased flow velocities and drag forces in the LVOT, pulling the anterior mitral leaflet toward the septum during systole, thereby mimicking the hemodynamics of obstructive HCM. Additional contributing factors may include changes in preload, afterload, and mitral valve morphology, including elongation or redundancy of the leaflets [2]. While the presence of SAM in the absence of LVH poses diagnostic and therapeutic challenges, it is important to recognize that this condition can resolve with reperfusion and improvement of regional myocardial contractility [3]. This case highlights a unique instance of reversible SAM in a patient with non-ST-elevation myocardial infarction (NSTEMI), apical akinesia, and no evidence of LVH, successfully managed with percutaneous coronary intervention (PCI).

## Case presentation

A 54-year-old female patient presented to the emergency department with atypical chest pain that began the day prior to admission. The pain was non-exertional, aggravated by sitting, and relieved when lying supine. She also reported palpitations but denied any fever, recent infections, or respiratory symptoms. Her medical history included essential hypertension, untreated dyslipidemia, and a family history of coronary artery disease. She had no history of smoking, alcohol, or drug use. Her regular medications included nicardipine 50 mg LP, venlafaxine 75 mg every other day, and amitriptyline 5 mg and alimemazine 10 mg at night.

Initial evaluation revealed stable vital signs: blood pressure was 100/67 mmHg, heart rate 75 bpm, and oxygen saturation 100% on room air. The physical examination was unremarkable, with no signs of heart failure. Laboratory tests showed elevated high-sensitivity troponin levels (264 ng/L, rising to 290 ng/L), while CRP and D-dimer were within normal limits. Electrocardiography revealed sinus rhythm with small Q waves in the inferior leads and lateral precordial leads, along with a 1 mm ST-segment elevation in lead II. Given the persistent chest pain and positive troponin, the patient was admitted to the coronary care unit for further management.

Cardiac magnetic resonance imaging (MRI) revealed regional wall-motion abnormalities with akinesia of the apical segments, along with compensatory hyperkinesia of the basal and mid segments (Video 1). The left ventricular ejection fraction was preserved at 56%. There was moderate mitral regurgitation (MR), potentially due to SAM of the mitral valve anterior leaflet, which resulted in a subaortic obstruction with a peak gradient of approximately 30 mmHg (Video 2). Right ventricular function and filling pressures were within normal limits.

**Video 1:** Cardiac MRI, axial view, subsequently confirmed an apical myocardial edema, indicating acute ischemia. MRI, magnetic resonance imaging.

**Video 2:** Cardiac MRI, sagittal view, demonstrating apical myocardial edema, indicating acute ischemia. MRI, magnetic resonance imaging.

A coronary angiogram performed on the same day revealed a monovessel coronary artery disease, with an intermediate lesion of the mid-left anterior descending artery (LAD), but left untreated.

Cardiac MRI subsequently confirmed an apical myocardial edema, indicating acute ischemia.

A second coronary angiogram was performed two days later, revealing a significant lipid-rich lesion in the mid-LAD involving the bifurcation with the first diagonal branch (Video 3). This repeat angiography was prompted by cardiac MRI findings, which demonstrated apical myocardial edema on T2-weighted sequences consistent with acute infarction. These imaging features raised suspicion for an ongoing Type 1 MI, warranting repeat evaluation of the coronary anatomy to guide definitive percutaneous intervention (Videos 3, 4).

**Video 3:** First coronary angiogram performed two days later demonstrating a significant lipid-rich lesion in the mid-LAD, involving the bifurcation with the first diagonal branch LAD, left anterior descending artery.

**Video 4:** Coronary angiogram post-angioplasty of the mid-LAD demonstrating significant lipid-rich lesion in the mid-LAD, involving the bifurcation with the first diagonal branch. LAD, left anterior descending artery.

This significant lipid-rich lesion in the mid-LAD was confirmed by optical coherence tomography (OCT), during which the mid-LAD lesion was treated with the implantation of a 3.0 x 28 mm XIENCE Skypoint drug-eluting stent (Abbott, Abbott Park, IL). OCT confirmed satisfactory stent expansion and apposition, with no dissection (Video 4). The diagonal branch was treated conservatively with balloon angioplasty using the POT-kissing-rePOT technique (Video 5). The initial diagnostic coronary angiogram was performed to evaluate the cause of NSTEMI. While a significant mid-LAD lesion was suspected, definitive stenting was deferred until further assessment of the infarct territory via cardiac MRI, which confirmed acute ischemia and apical myocardial edema consistent with Type 1 MI.

**Video 5:** OCT during which the mid-LAD lesion was treated with the implantation of a 3.0 x 28 mm XIENCE Skypoint drug-eluting stent. OCT confirmed satisfactory stent expansion and apposition, with no dissection. OCT, optical coherence tomography.

The patient’s clinical course was favorable. There were no arrhythmias or conduction disturbances during telemetry monitoring, and her chest pain did not recur.

The patient was discharged in stable condition on dual antiplatelet therapy (aspirin and ticagrelor), high-dose statin therapy (atorvastatin 80 mg), beta-blocker (bisoprolol 1.25 mg twice daily), an angiotensin-converting enzyme (ACE) inhibitor (ramipril 2.5 mg), and continued her psychotropic medications, including venlafaxine, amitriptyline, alimemazine, and aripiprazole. A transesophageal echocardiography (TEE) was planned to further evaluate mitral valve anatomy and function.

TEE after three weeks showed normalization of apical wall motion and left ventricular function (Video 6). The MR resolved, and the subaortic gradient was no longer significant.

**Video 6:** TEE after 3 weeks demonstrating normalization of apical wall motion and left ventricular function and resolution of the mitral regurgitation. TEE, transesophageal echocardiography.

In summary, this is a case of NSTEMI due to a significant mid-LAD lesion, successfully managed with drug-eluting stent implantation following initial diagnostic work-up with cardiac MRI. The patient's evolution was favorable, with full recovery of left ventricular function and resolution of SAM, most probably due to hyperkinetic basal segments in the absence of LVH. Left ventricular morphology was carefully assessed to exclude HCM. The left ventricle was non-dilated (end-diastolic diameter 45 mm) with normal wall thickness measurements, including an interventricular septal thickness of 8 mm and a lateral wall thickness of 7 mm, both below established echocardiographic thresholds for LVH. Left ventricular volumes were within normal limits (end-diastolic volume 115 mL; end-systolic volume 42 mL), and systolic function was preserved (ejection fraction 63%). No septal bulge, asymmetric hypertrophy, or structural abnormality capable of explaining fixed LVOT obstruction was identified on echocardiography or cardiac MRI, and consequently, resolution of the MR.

## Discussion

Pathophysiology of acquired SAM in ischemia

In this patient, acute apical myocardial dysfunction led to regional geometry changes that promoted SAM of the mitral valve despite the absence of LVH. The akinetic apical wall and hyperdynamic basal segments create a “ballooning” of the apex and vigorous contraction of the base, resulting in high flow velocities and Venturi/drag forces that tug the anterior mitral leaflet toward the septum. This distortion of LV shape and accentuated basal contractility after infarction produce dynamic LVOT obstruction [1,2]. In other words, preserved basal myocardium ejects forcefully into a reduced apical cavity, generating a pressure gradient that pulls the mitral valve anteriorly. This mechanism is analogous to that seen in Takotsubo stress cardiomyopathy, where basal hyperkinesis and mid-apical ballooning often coexist with SAM and acute MR [3]. Importantly, no chronic hypertrophy is required; acute ischemia and hypercontractile basal segments alone are sufficient to produce SAM via altered LV geometry and flow dynamics [1,2].

Diagnostic considerations and imaging modalities

Patients with SAM in the absence of HCM should undergo regular follow-up with echocardiography. These assessments may show either low- or high-pressure gradients across the LVOT, both of which can become significant under certain conditions, such as dehydration or a fast heart rate. It is also important to perform follow-up tests that include exercise or bedside maneuvers like the Valsalva maneuver, which can help reveal any signs of dynamic LVOT obstruction that might not be apparent at rest.

Initial TTE is crucial to demonstrate the regional wall-motion abnormalities (apical akinesis, basal hyperkinesis), mitral leaflet motion, and any LVOT gradient. In our case, TTE likely revealed an apical wall-motion defect with unexpected SAM and MR. TEE can therefore confirm leaflet morphology and measure the obstruction more accurately. For example, in one reported MI case, TEE showed the anterior leaflet striking a prominent septal bulge and causing severe MR with a dynamic gradient [1]. Echocardiography thus differentiates SAM from fixed mechanical causes (e.g., papillary rupture) and quantifies its hemodynamic significance.

Cardiac MRI can further delineate the etiology by characterizing tissue injury. In ambiguous cases, it can distinguish an ischemic infarction (with subendocardial or transmural late gadolinium enhancement (LGE)) from Takotsubo (with minimal/no LGE) or from HCM (diffuse fibrosis in hypertrophied segments) [3]. MRI also accurately maps the segments involved. Intracoronary imaging (OCT) may be used during PCI to confirm the morphology of the culprit plaque in the LAD lesion, although it does not directly visualize SAM. Together, multimodality imaging confirms that the obstruction is functional and acute rather than anatomic: lack of septal hypertrophy on echo/MRI, new wall-motion abnormalities matching the infarct territory, and resolution of SAM after reperfusion all point to an acquired phenomenon.

Distinguishing transient SAM from HCM

Transient SAM mimics HCM physiology but must be distinguished from true obstructive HCM. Key distinguishing features include the acuity of presentation and the absence of chronic structural disease. In HCM, SAM is usually associated with a hypertrophied septum or mitral apparatus abnormalities and is persistent; by contrast, in our patient, SAM appeared acutely with the infarct and resolved after revascularization, arguing against HCM. Echocardiography and MRI can confirm that septal thickness is normal and there is no asymmetric hypertrophy. Clinically, HCM patients often have a history of murmur or a family history of cardiomyopathy, whereas our patient had acute ischemia. Moreover, provocative maneuvers (e.g., Valsalva, exercise) exacerbate SAM in HCM, whereas here, SAM was linked to ischemia and basal hyperkinesis from stress. Thus, although the hemodynamics (LVOT gradient, MR) may appear identical, context and imaging distinguish transient ischemic SAM from genetic HCM. Clinically, patients with HCM often have a history of cardiac murmur or a family history of cardiomyopathy, whereas our patient presented with acute ischemia. Moreover, provocative maneuvers (e.g., Valsalva, exercise) typically exacerbate SAM in HCM, whereas in this case, SAM was temporally linked to ischemia-induced apical akinesia and compensatory basal hyperkinesis. Genetic testing for HCM was considered; however, it was not pursued given the absence of LVH on multimodality imaging, lack of family history suggestive of inherited cardiomyopathy, and complete resolution of SAM, LVOT obstruction, and MR following revascularization. Thus, although the hemodynamic consequences (LVOT gradient and MR) may appear similar, clinical context and imaging findings clearly distinguish transient ischemic SAM from genetic HCM.

Therapeutic implications and management

Management must address both the obstruction and its underlying cause. In acute SAM with dynamic LVOT obstruction, standard cardiogenic shock therapies (inotropes, intra-aortic balloon pump (IABP)) can worsen the gradient and should be used cautiously. Instead, treatment principles mirror those in Takotsubo LVOT obstruction: volume repletion and beta-blockade to reduce basal contractility and LVOT gradient [4,5]. For example, IV fluids and IV β-blockers can diminish hyperkinesis and limit SAM, whereas dobutamine or nitrates may aggravate it. Importantly, urgent revascularization by PCI addresses the root cause. Restoring flow to the LAD allows the apical myocardium to recover, which reverses the geometric distortion and abolishes SAM. In our case, successful PCI immediately relieved ischemia; within days, the wall motion normalized and SAM/MR resolved. Adjunctive medical therapy, such as beta-blockers (to temper residual basal hyperkinesis) and ACE inhibitors (to favorably remodel the ventricle), can be continued during recovery. This approach contrasts with HCM, where SAM is managed with chronic gradient reduction (sometimes with septal reduction therapy). Here, gradient reversal is achieved primarily by fixing the infarct.

Reversibility and prognosis

One of the hallmarks of ischemia-induced SAM is its reversibility. As regional wall motion recovers, the LV cavity returns toward normal geometry, and the Venturi forces abate, so SAM and MR resolve. This was seen in our patient: follow‑up echocardiography after several days showed no SAM or significant MR. Similar case series report that LVOT obstruction and SAM in acute coronary syndromes typically improve once infarcted myocardium recovers and compensatory basal hyperkinesis abates [6]. Dynamic SAM in acute MI does not imply permanent outflow obstruction or need for surgical intervention, unlike HCM. The transient nature of the lesion is a reassuring prognostic sign, provided revascularization is achieved. Clinicians should be aware that MR in this setting is often functional and may resolve, avoiding unnecessary mitral valve surgery.

Comparison with the literature

Our case shares features with several reports of LVOT obstruction in acute coronary syndromes, but also has unique aspects. Like the patient described by Aghajani et al., we observed apical akinesis with basal hyperkinesis causing SAM in the setting of acute anterior MI [2]. Others have reported similar dynamic obstruction following anterior STEMI or even during Takotsubo-like apical ballooning [4]. What is notable here is the occurrence in NSTEMI (rather than classic STEMI) and documentation with multimodality imaging (echocardiography plus OCT-guided PCI). Unlike many HCM-like presentations, our patient had no septal bulge or myocardial hypertrophy on imaging, indicating that the LVOT obstruction was not due to fixed structural remodeling but rather to a dynamic functional mechanism occurring on the background of an acute ischemic insult.

Importantly, while the LVOT obstruction itself was dynamic and reversible, it occurred in the setting of a significant structural coronary lesion of the mid-LAD requiring percutaneous revascularization. The rapid resolution of SAM after mid-LAD stenting emphasizes the central role of ischemia as the triggering substrate. In contrast to cases where SAM persisted despite maximal medical therapy (often complicated by cardiogenic shock, as in the IABP case above [1]), our patient improved once the culprit lesion was stented. This case thus reinforces reports that emergent PCI can itself be therapeutic for dynamic SAM, a point less emphasized in older literature.

## Conclusions

This case highlights a rare but clinically important phenomenon of transient SAM with LVOT obstruction secondary to acute ischemia in the absence of structural heart disease. Recognition of ischemia-induced SAM is essential in emergency and cardiology practice, as inappropriate use of inotropes or vasodilators may exacerbate LVOT obstruction, whereas beta-blockade, volume optimization, and prompt revascularization can facilitate rapid reversibility. The case underscores the importance of follow-up imaging to confirm resolution and to avoid misdiagnosis as HCM, which may otherwise lead to unnecessary long-term management. By illustrating a reversible, ischemia-driven mechanism of SAM without LVH, this report adds to the limited existing literature and highlights a diagnostic pitfall that remains underrecognized. Future studies are warranted to identify predictors of SAM reversibility following acute ischemia and to better define optimal management strategies. The conclusions are limited by the single-case nature of this report, emphasizing the need for larger observational studies.
